# Evaluating the costs and benefits of marsh-management strategies while accounting for uncertain sea-level rise and ecosystem response

**DOI:** 10.1371/journal.pone.0200368

**Published:** 2018-08-15

**Authors:** Marco Propato, Jonathan S. Clough, Amy Polaczyk

**Affiliations:** Warren Pinnacle Consulting, Inc., Waitsfield, Vermont, United States of America; University of Sydney, AUSTRALIA

## Abstract

Prioritization of marsh-management strategies is a difficult task as it requires a manager to evaluate the relative benefits of each strategy given uncertainty in future sea-level rise and in dynamic marsh response. A modeling framework to evaluate the costs and benefits of management strategies while accounting for both of these uncertainties has been developed. The base data for the tool are high-resolution uncertainty-analysis results from the SLAMM (Sea-Level Affecting Marshes Model) under different adaptive-management strategies. These results are combined with an ecosystem-valuation assessment from stakeholders. The SLAMM results and stakeholder values are linked together using “utility functions” that characterize the relationship between stakeholder values and geometric metrics such as “marsh area,” marsh edge,” or “marsh width.” The expected-value of each site’s ecosystem benefits can then be calculated and compared using estimated costs for each strategy. Estimates of optimal marsh-management strategies may then be produced, maximizing the “ecosystem benefits per estimated costs” ratio.

## Introduction

Conservation of coastal wetlands can provide a wide range of benefits to coastal communities, from increased resilience to storm events [1] to the provision of suitable habitats for animals and plants that are important ecologically and economically [2]. Tidal wetlands are capable of sequestering carbon and other nutrients [3], they also filter upland and runoff waters from pollutants and sediments and provide a protective buffer to reduce shoreline erosion due to wave action. Marsh and natural areas can also be important for their social, historical, and recreational role within coastal communities [4].

However, marsh areas have been degraded or lost as a result of human activities [5]. In addition, changes in climatic and ecological conditions and pressures from infrastructure development complicate effective conservation planning and management. For example, accelerating rates of sea-level-rise (SLR) require coastal managers to consider not only existing tidal flooding conditions, but also potential changes that may occur in the future. In particular, marshes can respond to increased inundation by migrating inland and colonizing areas that were previously at higher elevations [6] [7]. In many coastal communities, marsh migration can be complicated by the fact that land is not available because it is highly developed, or migration pathways are impeded by the presence of obstructions such as roads and other infrastructure [8].

Because of these factors, conservation planning and management under changing climate conditions, particularly sea level rise, can be complicated by the wealth of divergent data sets available and multiple policymaking goals. In addition, there may be several possible conservation and adaptation strategies whose costs and benefits should be evaluated while considering uncertainty both in future sea-level rise and dynamic marsh response. In this paper, a modeling framework, the Dynamic Marsh Management Tool (DMMT), is proposed to assist policymakers in planning and prioritizing coastal-marsh areas for adaptation and conservation strategies. This tool evaluates the costs and benefits of alternative management and adaptation strategies while accounting for environmental factors, socio-economic factors, and the potential protection of developed and undeveloped areas. In addition, changes of land cover due to sea-level rise are estimated by applying the stochastic version of the Sea Level Affecting Marshes Model (SLAMM) that allows land cover projections to be evaluated in terms of their likelihood of occurrence with respect to input-data and parameter uncertainties and uncertainty in future sea level rise. In depth examination of model limitations and effects of uncertainties on SLAMM land cover projections can be found in [9–11].

A key feature of the DMMT is that while the general architecture and framework is consistent across applications, stakeholder and expert input is critical in defining the most relevant ecosystem services, and their qualitative and quantitative evaluation can vary by agency or by evaluative task. The relative importance of each of the services is defined by the user of the tool (and can be redefined and re-run if desired). When integrated with time-varying SLAMM results, this tool provides a method to aggregate information in a meaningful and simple way while explicitly including model uncertainty as part of conservation planning and management.

The manuscript is organized as follows. A method section first describes the general modeling framework of DMMT, SLAMM projections and land cover uncertainty estimations. An application example in New York City (NYC) guides the reader through the different steps taken in model application, followed by a discussion section.

## Methods

**Fig 1:**
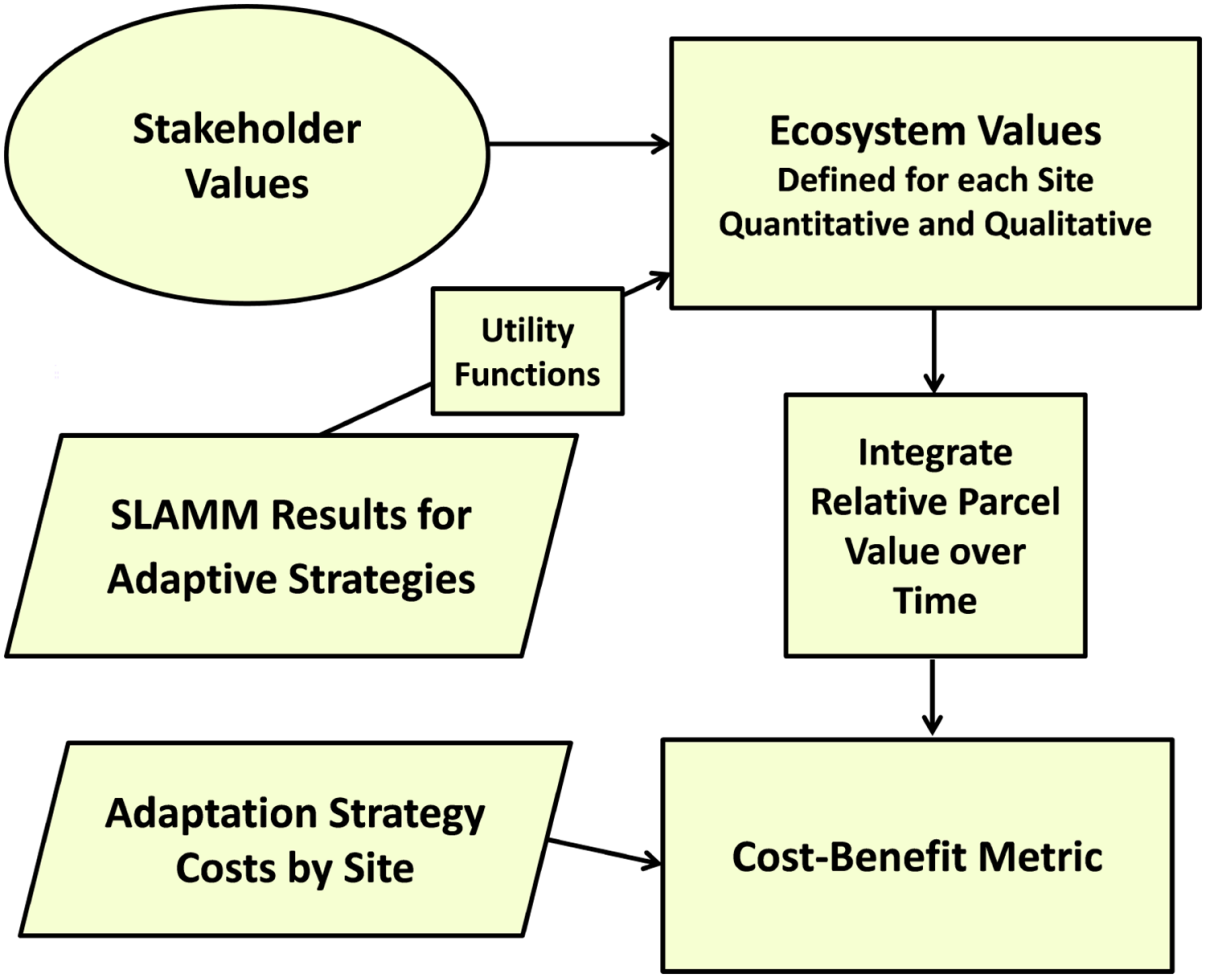
Detailed schematic for the Dynamic Marsh Management Tool.

This section describes the general framework of the DMMT including SLAMM model updates, and uncertainty projections. The project architecture is to (1) identify possible adaptation and management strategies to be implemented in the study area, (2) predict land-cover changes under SLR conditions for each of these strategies using SLAMM, (3) define and calculate ecosystem service values that allow the evaluation of the long term benefits of each strategy; (4) estimate costs associated to the implementation of each adaptation strategy; and finally (5) integrate land cover projections, ecosystem service values, and stakeholders inputs to identify the optimum management action that maximizes future benefits while being cost effective (Fig 1). A key component of this project is direct stakeholders participation in identifying and characterizing these services and providing their relative importance in the decision making process.

One key aspect of this analysis is the integration of uncertainties in model inputs and SLR scenarios. Further, when evaluating ecosystem values, not only ecocentric but also human-centered services are accounted for when quantifying future benefits. Rather than a “black box” linking model outputs to benefits, the proposed approach is transparent and modifiable by stakeholders. The approach therefore comprises three significant features: end-user engagement, the capability to account for local expert knowledge, and the incorporation of wetland ecosystem-service features that may be more qualitative and difficult to quantify.

### Adaptation strategies

In general, appropriate adaptation strategies for marsh conservation under SLR conditions depend on the study area and stakeholders interests.

Typically, allowing marsh transgression to private or public land is one considered strategy. For these cases, planning involves land-parcel acquisition, transfer, or easement and often also restoration of land areas that are currently developed (e.g. removing man made barriers such as concrete to facilitate marsh establishment).

Thin-layer deposition of dredge material on low marshes (regularly-flooded marshes) to add elevation capital is a strategy that has been closely studied in recent years [12]. However its long term benefits under SLR are not fully understood.

Other adaptation strategies that may be considered are restoration of tidal flows for marsh systems that are currently tidally restricted because of the presence of man-made restrictions such as culverts or bridges. Similarly, restoration of tidal flows may be examined in areas that are currently covered by open water because of the presence of dams or other tidal impediments.

Some additional strategies may be very specific to the study area. For example, for NYC this study examined an adaptation strategy that considers restoration of marsh edges to their 1970 extents (marshes have historically constricted landward in this study area). This may be economically competitive due to the high cost of land in the study area. Despite the costs of marsh restoration, moving toward the open water is being considered as no land purchase would be required.

To better clarify these concepts, the five adaptation strategies considered for the New York City case study are described below. These were:

- **Protect dry land**: This strategy assumes that land owners will armor shorelines to prevent marsh migration. With this option no marsh transgression into dry land is allowed to occur and all ecosystem benefits must accrue from existing marsh boundaries. This is considered the “no-action” case in terms of marsh management because it only looks at marsh projections of existing marsh footprints with no action on it. For comparison to other adaptation scenarios, this may be considered the “base case” as ecosystem-service benefits are the lowest.
- **Acquisition/transfer of undeveloped parcels**: This strategy assumes that marsh migration will be allowed onto undeveloped dry land. The cost estimate for this strategy is based on the purchase of land parcels or easements from landowners.
- **Acquisition/transfer of developed parcels and restoration**: Similar to the undeveloped strategy above, this strategy also includes the purchase of developed lands and their physical restoration to marsh habitats.
- **Restoration of marsh edges to 1970’s marsh footprints**: This strategy assumes that wetland areas identified in 1974 wetland maps, but that today are open water or tidal flat, will be restored to marsh lands. Restoration is assumed to restore elevations for these new marsh areas to mean tide level.
- **Thin-layer deposition**: This strategy considers the deposition of dredge material on low marshes (regularly-flooded marshes) to add elevation capital. To mimic this practice, the elevation layer was modified by adding 20 cm to the elevations of low marsh areas that were within 60 m distance from open water or dry land, which was the assumed distance that could be reached by a high-pressure sprayer from a barge or a truck.

### SLAMM projections

Projections of land-cover changes under each adaptation scenario and SLR condition are produced using SLAMM. SLAMM is widely recognized as an effective model to study and predict wetland response to long-term sea-level rise [13]. The model has been applied in every coastal US state [14–20]. Uncertainties in the future rate of SLR and the dynamic nature of marsh response to SLR can obscure straightforward predictions of wetland fate. The stochastic version of SLAMM accounts for these uncertainties by representing all model parameters and driving variables as distributions; errors in spatial data are represented using a spatially-autocorrelated uncertainty/error field [21].

A recent development within the model is the capability to include high resolution roads and infrastructure data (as lines or points with associated elevations) to better account for the effects that infrastructure has on marsh-inundation pathways (especially roads). This new SLAMM module also calculates the frequency of flooding of roads and infrastructure from combined SLR and storm surge.

Another model update is an attempt to account for the potential loss of elevation capital that occurs when irregularly-flooded marsh is converted to regularly-flooded marsh or when regularly-flooded marsh is converted to tidal flat. Changes in pore-water salinity are known to cause changes in bacterial composition and this can result in rapid decomposition of underground biomass [22]. Marsh collapse has been observed in marsh systems when land-cover conversions occur [23]. Within the SLAMM marsh-fate model, marsh-loss transitions include corresponding elevation losses based on data collected by Dr. David Burdick and his team at the University of New Hampshire [24,25]. This team observed that the average loss of elevation capital when irregularly-flooded marsh converts to regularly-flooded is around 7 cm, while from regularly-flooded marsh to tidal flat is 19 cm. One preliminary observation from recent SLAMM simulations including this marsh collapse mechanism, is that loss of elevation may be partially offset by marsh accretion feedbacks that cause marshes at lower elevation to horizontally accrete more inorganic material and move vertically more rapidly [26]. Because of this feedback, our finding is that the marsh-collapse addition to SLAMM causes only minor site-specific differences in marsh-fate predictions under moderate SLR scenarios, and does not have a significant effect on landscape-level predictions. Uncertainty in measured marsh-collapse rates have also been incorporated in the model’s uncertainty analysis. This is especially relevant as site specific marsh-collapse data are often not available. Therefore one can assign uncertainty distributions with wide ranges to account for the lack of precise knowledge regarding these parameters.

As noted above, all of the site-specific data required by SLAMM, such as the spatial distribution of elevations, wetland coverages, tidal ranges, accretion and erosion rates, sea-level rise and subsidence rates, are affected by uncertainties that can propagate into the predicted outputs. For each of the model input parameters, uncertainty distributions were derived based on available site-specific data and feasible bounds of the variable considered. Distributions were derived reflecting the potential for measurement errors, uncertainty within measured central tendencies, and professional judgment [27]. The SLAMM uncertainty estimation module then employs a Monte Carlo approach in which the model is run hundreds of times over different input parameters, simultaneously drawn from their uncertainty distributions using efficient Latin-Hypercube sampling. Results are assembled into probability distributions of estimated land coverages [10].

### Ecosystem services evaluation with stakeholder participation

Several ecosystem services may be considered in conservation planning and management when assessing the general benefit provided by a particular marsh system and its surrounding areas. Ecosystem services can be nature-centered, such as nekton and bird habitat preservation or nutrient sequestration capability, or human-centered, such as providing areas for recreation or storm wave energy reduction to protect population and infrastructure. The set of services and their relative importance depends on stakeholders/expert/agency input, priorities or mission. The ensemble of ecosystem services selected forms the basis for quantifying management benefits and thus evaluating alternative adaptation strategies.

To quantify the benefits of each ecosystem service, it is necessary to identify key variables and their relationship with the service. Generally, ecosystem-service values can be related to quantitative variables that may be estimated by a model, such as land-cover type, land-cover extent, width, edge, or fragmentation. For example, nutrient sequestration capacity depends both on the marsh type and the marsh extent covering a given area. However, landscape features may not be the only properties characterizing ecosystem services. A marsh that is buffering a highly populated area may have a higher wave-attenuation value than an identical one that is located in a relatively undeveloped area. Additionally, there are ecosystem-service values that are difficult to assess quantitatively, such as cultural, recreational, and political values. For example, the high recreational use of a particular marsh area may be explained by proximity to public transportation or other factors outside of the domain of the models. As described below, the estimation of these ecosystem-service components relies on input from experts and stakeholders.

The mathematical form of each ecosystem-service value *S* is defined here as:

S=F(Landcoverfeatures)*V (1)

Where *F* is a function of landscape features and *V* is a function accounting for other quantitative variables (when available) and/or stakeholder inputs. *V* could also be a function of land cover features (i.e. land cover type) but here it is assumed to be dependent only on the land parcel as a simplification.

In general, under sea level rise conditions, the ecosystem service value *S* is variable in time as land cover changes as a result of increased inundation. The exact mathematical form of the ecosystem service value *S* may be complicated and quite often unknown. Relationships between ecosystem functions (which are maintained by an ecosystem whether humans are there or not) and services provided as result of human use and interactions with the ecosystem may be non-linear, have thresholds, may be negative (i.e. ecosystem disservice) or other complicated forms. For example a land parcel may provide recreational activities service only if there is a minimum quantity or location of undeveloped dry land area available, but below that threshold area the service is not significantly provided. Furthermore, key landscape-feature inputs may also differ between services (area, type, width, and other landscape measurements). As discussed in more details below, the DMMT is flexible to define these mathematical relationships on a case-by-case basis. Literature and stakeholders inputs are used to define the specific functional forms and variables that quantify the service for a particular study area. Below a simple example of service quantification is provided.

#### Landcover ecosystem service values

Consider the nutrient sequestration capacity estimation of a marsh parcel. From the literature, e.g. [3], a simple linear model can be developed:

$$F\;=\;s_{1}A_{1}+s_{2}A_{2}+\cdots +s_{n}A_{n}$$ (2)

Where each term *A*_i_ is the area occupied by land cover class *i* and *s*_*i*_ is the nutrient accumulation rate per unit area of land cover type *i*. The nutrient accumulation rates are generally positive. However, these coefficients could also be negative when considering methane, ammonium, and nitrous oxide releases. In practice, only fresh and salt marshes contribute to the overall nutrient sequestration.

This model says that as more marsh area that is available in a marsh parcel, more nutrients are sequestered. However, there may be local factors that may influence nutrient capacity other than marsh coverage area. For example, health condition of a marsh system may significantly influence nutrient sequestration or similarly, proximity to an agricultural area or a golf course where higher than normal nutrients concentrations may yield to more nutrients to sequester. These more local and qualitative factors may be accounted in the *V* function.

#### Qualitative and stakeholder ecosystem service values

As described by Eq (1), *V* is the function accounting for other important factors affecting ecosystem service values. These factors may be quantified using spatial data when available; for example nutrient sequestration service values can be updated using available marsh-condition indices. When these data are not readily available expert knowledge and stakeholder inputs may be used to provide an estimate of *V*. As further described below, since ecosystem services provided by each land parcels are compared to one another, *V* needs to quantify the *relative* (higher or lower) effectiveness in providing the service. Following the previous example, if two parcels have similar marsh coverages then initially they provide similar nutrient sequestration, estimated by Eq (2). However, experts/stakeholders may suggest that one unit area of marsh provides double the service at one location compared to the other. In this case, one would set *V* equal to 2.0 and 1.0 (or 20 and 10,…). respectively to account for this relative difference in providing the service. In principle *V* may be variable in time. However, the temporal variability for this ecosystem value component is difficult to assess (e.g. how marsh condition indices change over time is unknown). For simplicity in this application, the component *V* is assumed constant over time.

#### Overall ecosystem service benefit

A manager’s overall objective is to identify the proper adaptation strategies across land parcels considered that maximize all ecosystem-service benefits. However, in general, it is difficult, sometimes impossible; to identify an optimal policy that simultaneously maximizes all ecosystem services.

One of the most common methods for multi-objective problems is to define a global ecosystem benefit value function in which all services are combined to form a single function. For each land parcel, the global ecosystem service value *W* is defined as:

$$W\;=\;\sum_{i=1}^{m}w_{i}S_{i}$$ (3)

Where *w*_*i*_ is the weight reflecting the importance of each ecosystem service *S*_*i*_ defined in Eq (1) and *m* is the total number of services considered. In general, these weights are identified by the decision maker and stakeholders. An iterative Delphi survey process is one way to proceed to allow users to record their preferences and to understand how their survey results affect the tool [28]. Survey results (and the reason that they were chosen) are made transparent after each round of the survey.

#### Ecosystem service normalization

The formulation above has the limit that the overall benefit *W* greatly depends on the range of the ecosystem services (e.g. a service whose values are an order of magnitude greater than another provides on overall benefit that is on order of magnitude greater). To overcome this problem each ecosystem service function *S* is scaled to obtain non-dimensional quantities:

$${\tilde{S}}_{i}\;=\;\frac{S_{i}}{\langle S_{i}(0)\rangle }\;with\;i\;=\;1,\ldots ,m$$ (4)

where 〈*S*_*i*_ (0)〉 is the initial average ecosystem value across all sites.

If the ecosystem service weights *w*_*k*_ are chosen such that *∑*_*i*_ *w*_*i*_ = 1, then the initial-condition rescaled ecosystem value *W* will be on average equal to 1. Parcels with *W* > 1 initially provide an overall benefit that is greater than the average, while the opposite is true for parcels with *W* < 1.

#### Accounting for time changes

Since land cover is projected to change over time as a result of SLR, planning can also consider time horizons and the importance of different planning dates in the decision making process by defining the time weighted global ecosystem benefit *Q* as

$$Q\;=\;\sum_{k=1}^{T}q_{k}W_{k}$$ (5)

where *q*_*k*_ is the weight assigned to time step *k* and *T* is the total number of time steps considered. The weight *q*_*k*_ may be a discount factor if a higher weight is desired for current conditions. The objective is to maximize the overall ecosystem service benefit *Q* while accounting for future projections, with assigned weights reflecting their importance in the overall planning time horizon.

#### Accounting for uncertainty

Finally, as future land cover projections are affected by uncertainty, in order to account for them in the planning process, the average land-cover results from Monte Carlo simulations are considered:

$$Z\;=\;\sum_{j=1}^{N}z_{j}Q_{j}$$ (6)

where in general *z*_*j*_ is the weight given to the *j*-th realization of total *N* Monte Carlo projections. This weight could be constant across all model realizations when all of them are assumed to occur with equal probability, or may reflect some additional knowledge of the likelihood of their occurrence.

### Adaptation strategy cost assessment

One key component of the DMMT is the assessment of costs associated with each adaptation strategy for each land parcel considered. These cost estimations may be complex when considering land-parcel cost, predicted inundated areas and physical activities associated with restoration interventions (such as removing concrete or deposit dredge material). A detailed description of cost estimations performed for NYC follows; this could be used as an example to replicate cost estimations in other study areas.

For New York City, cost estimates for adaptation strategies were produced with the significant assistance of NYC Parks. Costs were aggregated across each defined wetland site. For migration onto adjacent land the extent and location of the marsh-migration footprint was determined using SLAMM uncertainty analyses. Next, this land was broken into four categories: “NYC Parks owned,” “other public land,” “private developed land,” and “private undeveloped land.” Undeveloped land owned by NYC Parks was assumed not to have an associated marsh-migration cost. For other categories, detailed high and low estimates of costs for land transfer, land acquisition, or purchase of land easements were developed [29]. For this case study, the mid-point estimate of costs between the high and low estimate was used.

To calculate the cost of thin-layer deposition, the total surface area of regularly-flooded marsh available for thin-layer deposition at each site was calculated. This was then multiplied by a cost estimate per acre (0.4047 ha) to apply 20 cm of dredge material onto the marsh lands. The estimate of approximately $550,000 per acre ($1.36 million per ha) was developed by NYC Parks and includes costs for stabilizing the construction entrance, erosion control materials, waterfowl barriers, sand placement, plug planting, permitting, and project management and engineering [29]. The cost of marsh-edge restoration was estimated by NYC Parks to be $624,000 per acre ($1.54 million per ha) based on a similar set of calculations and estimates.

Finally, land-purchase costs were increased by 20% to account for market inefficiencies (i.e. you cannot exclusively purchase land converting to wetlands).

### Dynamic Marsh Management Tool

The DMMT is a Microsoft Excel-based tool with a Visual Basic computational engine that integrates and analyzes SLAMM uncertainty simulations for different adaptation strategies and costs, ecosystem service evaluations and stakeholders inputs to identify the optimal strategies and land parcels. A complete User’s Guide is available and a video-enhanced tutorial has been produced to help users understand how to use the tool. In addition, a guide to add new SLAMM uncertainty-analysis results into the DMMT has been produced. All of these materials may be found on the Dryad digital repository along with the DMMT case-study spreadsheets and their underlying source code: (doi:10.5061/dryad.6dq3r10).

### Case study results

This paper focuses on the case study developed for New York City study areas, though the DMMT has been also applied in Nassau County, NY, Suffolk county, NY and Casco Bay in Maine.

#### SLAMM base model and calibration

The starting point for model simulations for this project was a 5-m resolution simulation of coastal New York created in 2014 with NYSERDA funding [26]. The original model was then updated with elevation data that were of higher resolution and vertical accuracy as well with high resolution roads elevation data (rod elevations were assumed as average elevation of 1m buffer area around road centerline).

The model calibration was also updated to account for newer tide-range, accretion, and elevation data. Calibration results were consistent with previous model with an improved description of water flows and land cover agreement with tidal inundation. Please see the 2014 NYSERDA report [26] for a more detailed description of model calibration in this study area.

Only minimal changes were made to the original model parameters. The full set of SLAMM base model results in deterministic (individual sea-level rise scenarios) and uncertainty mode results may be found at the project website (warrenpinnacle.com/prof/SLAMM/NYSERDA2015). This site also includes a set of road vulnerability shapefiles with road inundation statuses estimated for each five-meter segment of road in the study area.

#### SLAMM adaptation strategies projections

As previously described, SLAMM uncertainty simulations were run for five adaptation strategies: No marsh migration (base case focusing only on existing marsh footprint), marsh migration to undeveloped land, marsh migration to developed land (land restoration also required), thin-layer deposition, and marsh edge restoration to 1970 footprints.

**Fig 2:**
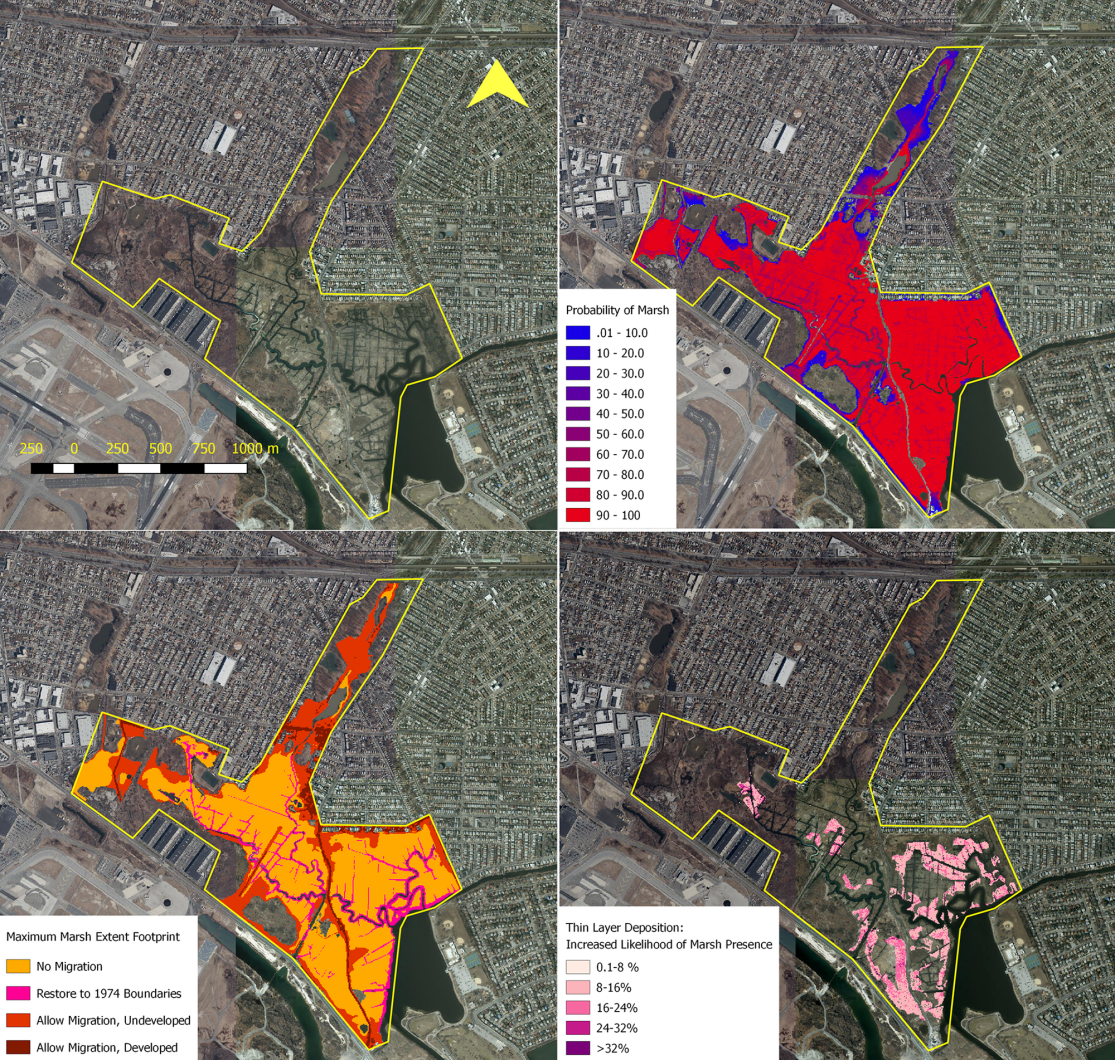
Upper left: Satellite imagery of Idlewild marsh parcels; Upper right: The maximum probability of marsh existence in 2085 given combined adaptation strategies; Lower left: The footprint of marsh existence in 2085 for each adaptation strategy; Lower right: The incremental benefit of thin-layer deposition at this site (the increased likelihood of marsh presence in the year 2085 following deposition). Satellite images: New York State Digitally Enhanced Orthoimagery from NYS GIS clearinghouse.

An example of these model applications may be found in Fig 2.

The upper left map in Fig 2 shows the satellite image for Idlewild marsh parcel. This marsh system is just north-east of the JFK International Airport. The lower left picture illustrates the combined potential marsh footprint by 2085 for each adaptation strategy considered. This map shows that the marsh system has good opportunities to increase its extent by allowing marsh migration in areas that today are not regularly inundated but that are expected to become inundated in the future, or by restoring the 1974 marsh boundaries.

However, uncertainty-simulation results not only provide information about what the marsh extent *may* be in the future but more importantly, probability maps are produced to inform how *likely* these areas are expected to be marsh in 2085 considering model uncertainties and the wide range of possible SLR scenarios, as shown in the upper right map. The likelihood for an area to be a marsh in the future is accounted in the application of the DMMT. For example, ecosystem benefits provided by blue (low probability) areas in the probability map are less than the ones from red (high probability) areas. The lower right map shows the increment in the probability of marsh existence by 2085 as the result of a single thin layer deposition application.

#### Ecosystem service values determination

**Fig 3:**
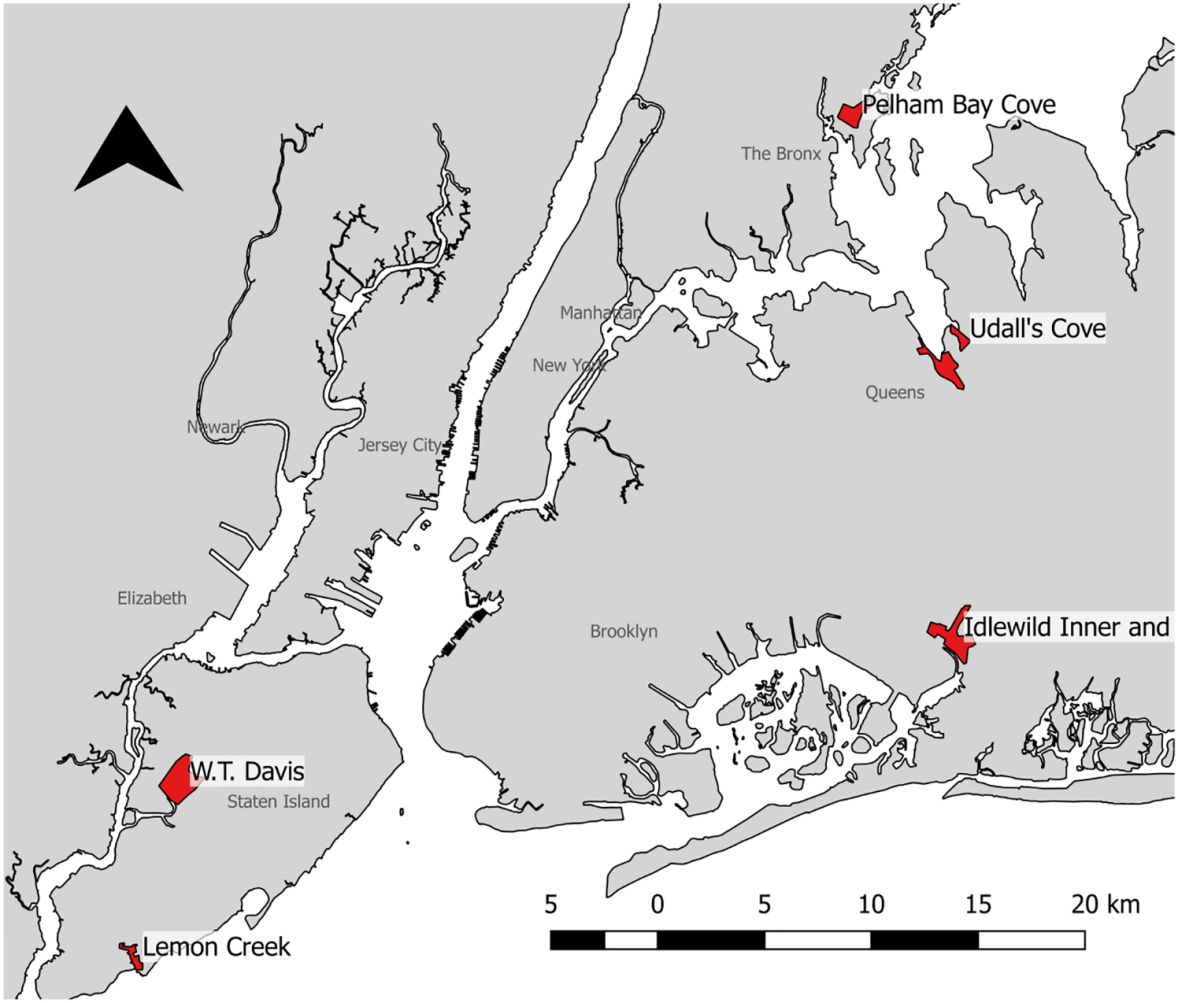
Six wetland sites selected for New York City case study. Shoreline data from NOAA (National Ocean Service Coastline of the Northeastern United States).

To complete this project, a team of stakeholders and experts was convened to define and provide feedback on several topics. This team consisted of professionals from New York City Parks, New York Department of Environmental Conservation, The Nature Conservancy, and NYC Planning among other groups. Six sites with wetlands were selected by stakeholders for decision-support analysis in the New York City study area (see Fig 3).

- Idlewild Inner and Outer
- Alley Creek, Queens
- Lemon Creek, Staten Island
- Pelham Bay Cove
- W.T. Davis
- Udall’s Cove, Queens

Next, the stakeholder team developed an “ecosystem services list,” or the complete list of ecosystem service values *S*_*i*_. The ecosystem services that were defined for this project were:

- Nutrient sequestration (C, N, P)
- Recreation
  - Undeveloped dry land
  - Wetland
  - Natural services to under-served communities
  - Nekton habitat proximate to fishing areas
- Habitat
  - Nekton habitat
  - Habitat connectivity
  - Bird habitat
- Wave attenuation/Flood damage reduction
- Political/Cultural/Historic value
- General preservation of natural areas

In general ecosystem services can be loosely defined. For example, the recreational function of a wetland can have several meanings for stakeholders: its presence can provide an area for bird watching, fishing, or other recreational activities that otherwise would not be possible is the marsh system was not there. It is the stakeholders that define the service and its importance.

Other services may be specific to a particular study area. For example, the presence of a natural area in particular locations of NYC accomplishes the mission of NYC Parks to provide recreational space for underserved neighborhoods.

Marsh ecosystem functions, such as the provision of Nekton habitat, may serve different purposes. Recreational fishing benefits from nekton habitat. Additionally, in itself, the nekton habitat has an important ecological value. Stakeholders define their relative importance: a fisherman may find the first more important while an ecologist will focus more on the second service.

Finally some ecosystem services can increase under conditions where other services decline. For example the “dry-land recreation” service of a particular land parcel may decrease as sea level rises while the recreation service provided by the marsh may increase at the same time due to marsh transgression over the dry-land area.

The third area in which stakeholders provided input was the definition of “utility functions.” Utility functions provide the relationship between “ecosystem services” and the quantity of marshes, marsh types, or other geometric metrics Eq (1). Some of the geometric metrics that were extracted from land-cover results for each defined marsh parcel were as follows:

- The predicted land-cover area for all SLAMM land-cover categories including regularly-flooded marsh, irregularly-flooded marsh, and tidal flats.
- The marsh to open water interface (meters). This interface is considered especially important habitat for nekton as compared to interior marsh [2].
- The “marsh width” perpendicular to shore (meters). This can assist in estimating the wave-attenuation benefits that a marsh may provide.

Stakeholders also provided the relative value that they have for each ecosystem services (*w*_*i*_ for each *S*_*i*_). DMMT can identify optimal actions given each individual user’s ranking (set of *w*_*i*_) or an aggregate of all stakeholders polled can be utilized. For the New York City study area, the priorities were defined as shown in Table 1. Habitat connectivity and fragmentation had the highest overall relative values. However, stakeholders emphasized the importance of carbon and nutrient sequestration as well; combining these three ecosystem services provided 23% of the ecosystem-service valuations.

**Table 1:** Relative ranking of ecosystem services based on NYC stakeholders survey.

Please provide the relative ranks for how important each of the following ecosystem services are to your decision-making process.	
Carbon Sequestration	7.6%
Nitrogen Sequestration	8.2%
Phosphorus Sequestration	7.5%
Undeveloped dry land recreation utility	4.5%
Marsh land recreation utility	4.5%
Natural areas for underserved communities	8.1%
Nekton habitat	13.6%
Habitat connectivity/Fragmentation	17.7%
Flood protection	14.0%
Political/Cultural/Historic value	5.5%
General preservation of natural areas	8.7%

Last, stakeholders were polled to quantify the site-specific strengths and weaknesses for each site relative to each ecosystem service identified (*V*_*i*_ associated with each *S*_*i*_). This quantity informs the question “which sites currently offer more or less of specific services based on their location and the health of the ecosystems?” For example, a marsh that is not proximate to developed lands offers fewer wave attenuation benefits than one which is directly protecting valuable infrastructure. A marsh with low stem density might be expected to sequester less carbon or nutrients than the same surface area of a thriving, higher-density marsh (that generates more biomass and traps additional sediment [30]). Spatial data and expert feedback can also be used to characterize these site-specific differences.

#### DMMT application

The DMMT combines these uncertainty results with the costs associated with each adaptation strategy to inform planners and managers as to where and how an available budget for marsh conservation can be used in the most beneficial way considering future landscape changes.

An intermediate result produced by the DMMT is a graph of “persistent marsh” per million dollars spent. Persistent marsh is defined as the surface area of marsh that will persist in the study area through the year 2100 as compared to the “no action” (protect dry land) scenario. A marsh that is predicted to persist for only 10 years will have one eighth the value of a marsh that is predicted to persist for the next 80 years.

**Fig 4:**
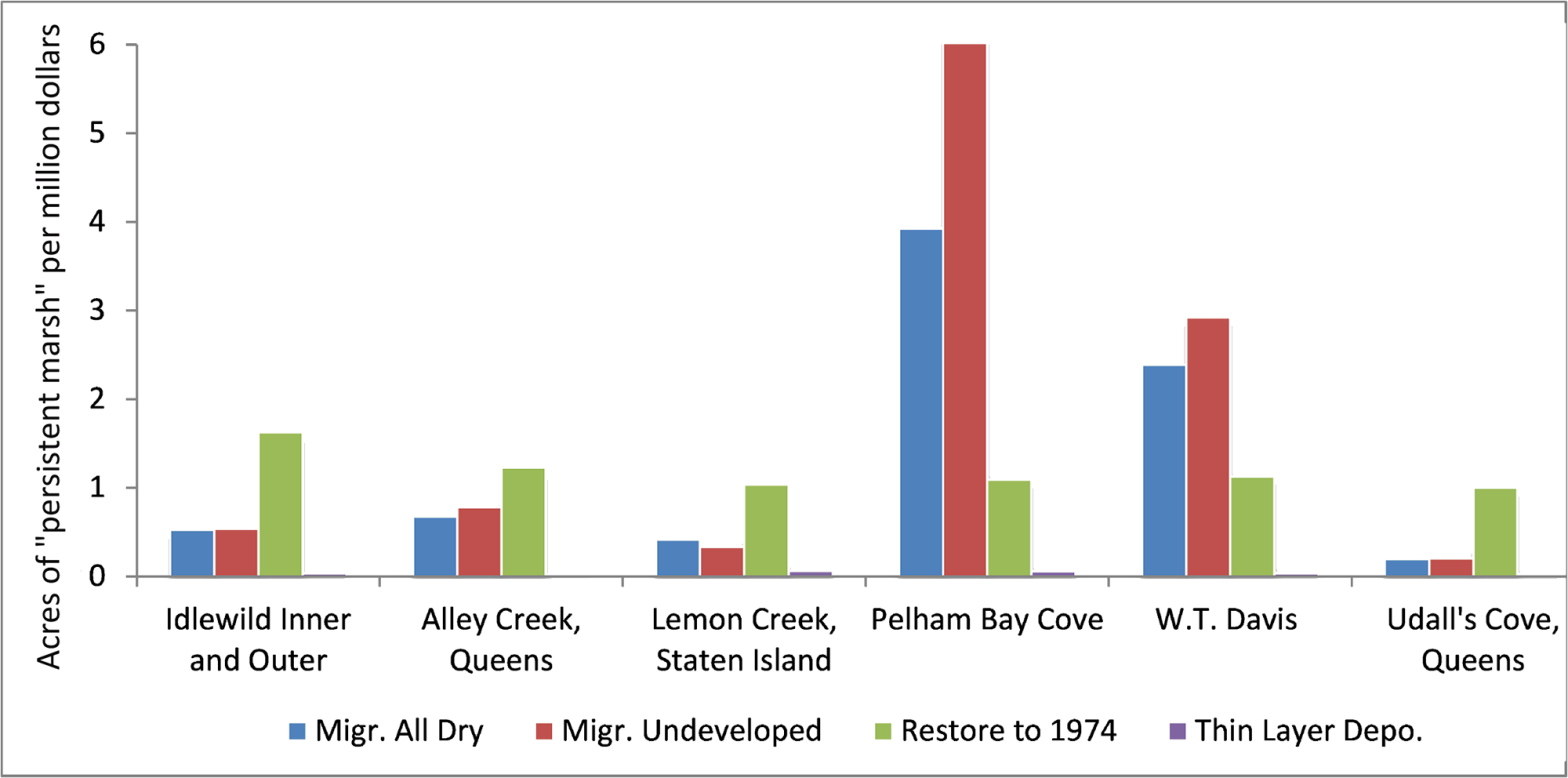
Acres of “persistent marsh” per million dollars spent. (These figures were provided in acres at stakeholder request; 1.0 acres = 0.4047 ha).

Within this study area, the most cost-effective actions are to allow marsh migration in Pelham Bay and at W.T. Davis parcels (Fig 4). The high quantity of public and NYC-Parks owned lands at these sites drive the low costs of these actions. For other sites, marsh restoration becomes the most cost-effective alternative due to the higher land costs estimated at these locations. This finding is consistent with those of Feagin and coworkers [4] who found that wetland survival is likely dependent on “the rate of return on property and housing investments” in lands adjacent to existing wetlands.

**Fig 5:**
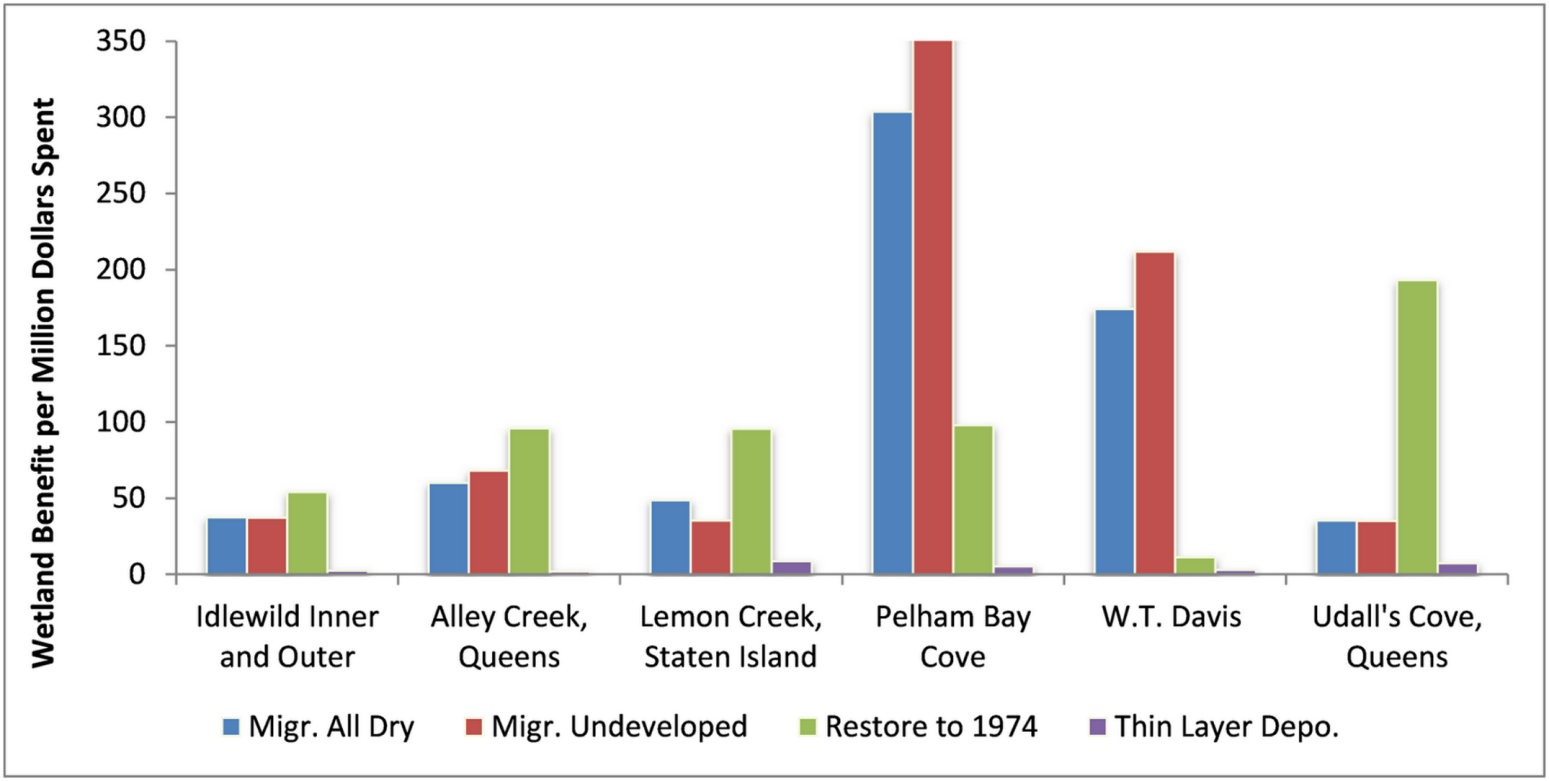
Aggregated wetland benefits predicted per million dollars spent (2016 to 2100).

While “areas of persistent marsh” created by each action is an interesting metric, one central assumption within the DMMT, is that not all marsh surface areas have equal value to stakeholders. When stakeholder preferences are incorporated into the DMMT as characterized in the survey-results above, a different set of optimal management actions are predicted. Fig 5 is similar to Fig 4 but it graphs the normalized wetland benefits “*W”* through 2100 per million dollars spent as opposed to persistent marsh surface area. The low cost of land at Pelham Bay Cove and W.T. Davis still makes marsh migration in those locations cost effective. Marsh restoration at Udall’s Cove becomes much more competitive, however, and W.T. Davis has now become the least cost-effective location for marsh restoration.

**Fig 6:**
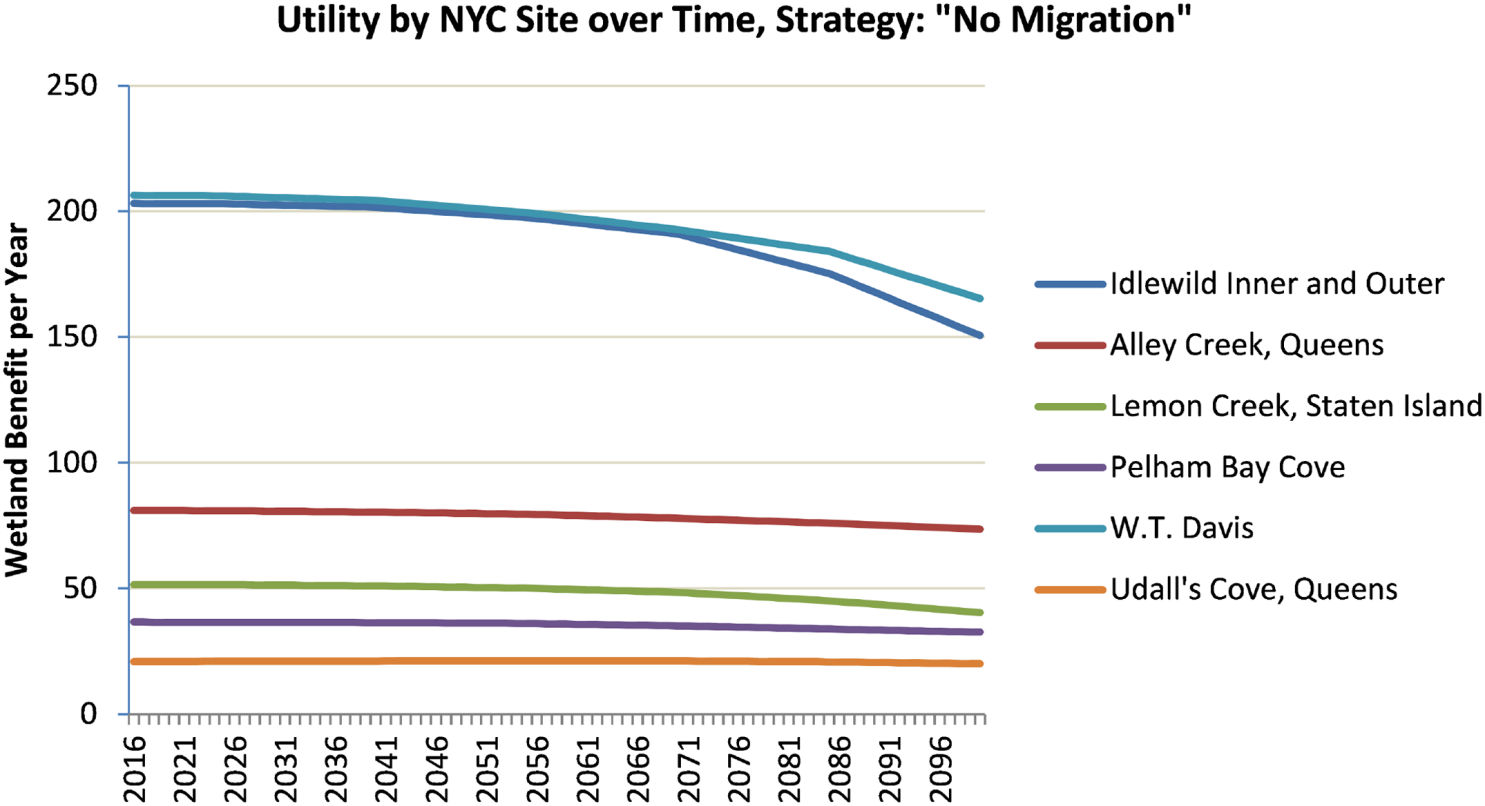
Time series of each NYC site’s utility under no-action scenario.

The DMMT allows the user to “drill down” into the cause of the types of results shown above, having numerous customizable graphs within its Excel-based interface. For example, Fig 6 shows the expected-value utility “*W”* for each site in the study area over time given uncertainty in future SLR. (In this figure, the initial condition utility “*W”* is normalized to show an average utility of 100 units at the simulation start time.) Not surprisingly, the two sites with the largest marsh surface area provide the largest quantity of ecosystem benefits at the start of the simulation, but notably they suffer the largest potential losses under sea-level rise as well. This figure can be reproduced to show time series for any of the alternative management strategies included in the model (e.g. marsh migration, marsh restoration, or thin layer deposition).

**Fig 7:**
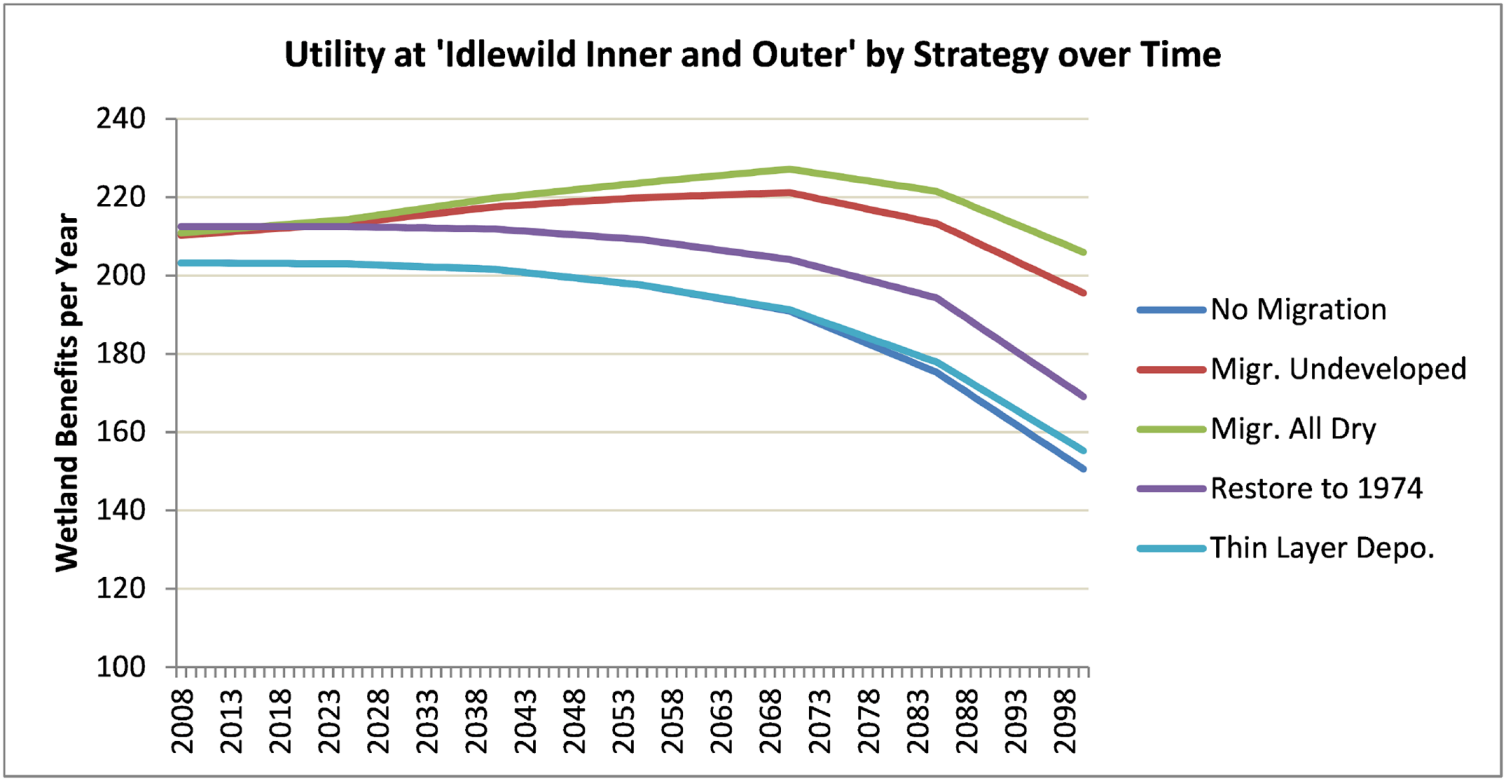
Time-series utilities estimated for the Idlewild marsh system.

Fig 7 shows the same set of expected-value utilities, but shows a single site comparing all the different adaptation strategies modeled. This figure suggests that allowing marsh to migrate inland provides potential increases in ecosystem benefits despite sea-level rise.

**Fig 8:**
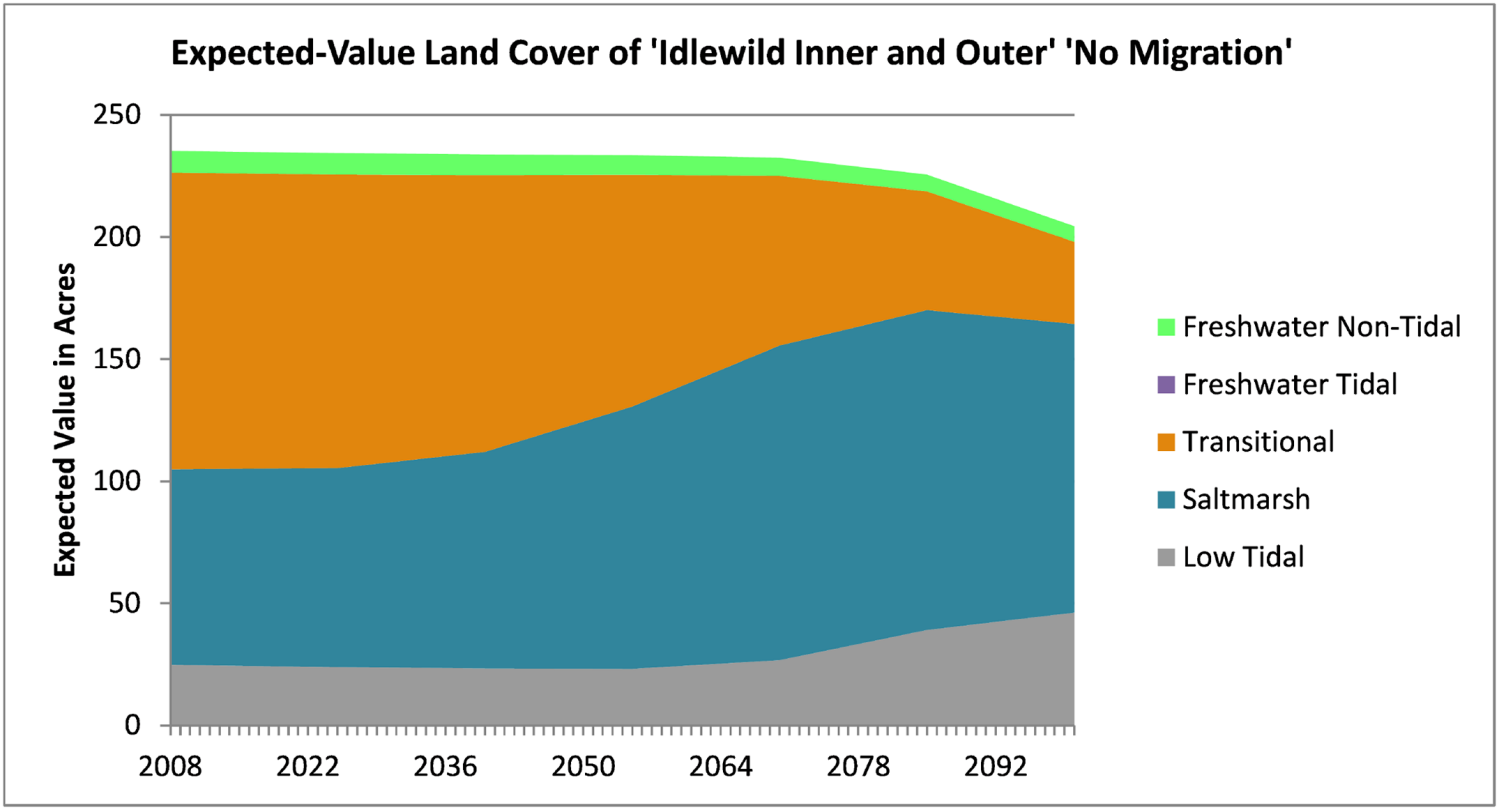
Expected-value aggregated land cover over time for the Idlewild marsh system. (these figures are provided in acres at stakeholder request, 1.0 acres = 0.4047 ha).

To further understand the data behind model predictions, expected-value land-cover predictions may be created for each site and each adaptation strategy modeled. (The 26 SLAMM Land-cover categories are combined into simplified categories to make the figure more readable). Fig 8 shows that the Idlewild marsh system is predicted to lose existing marsh lands overall and some of the irregularly-flooded marshes (“transitional” aggregated category) are predicted to be converted to regularly-flooded marshes (the “saltmarsh” aggregated category). The “low-tidal” category consists of non-vegetated beaches and tidal flats.

**Fig 9:**
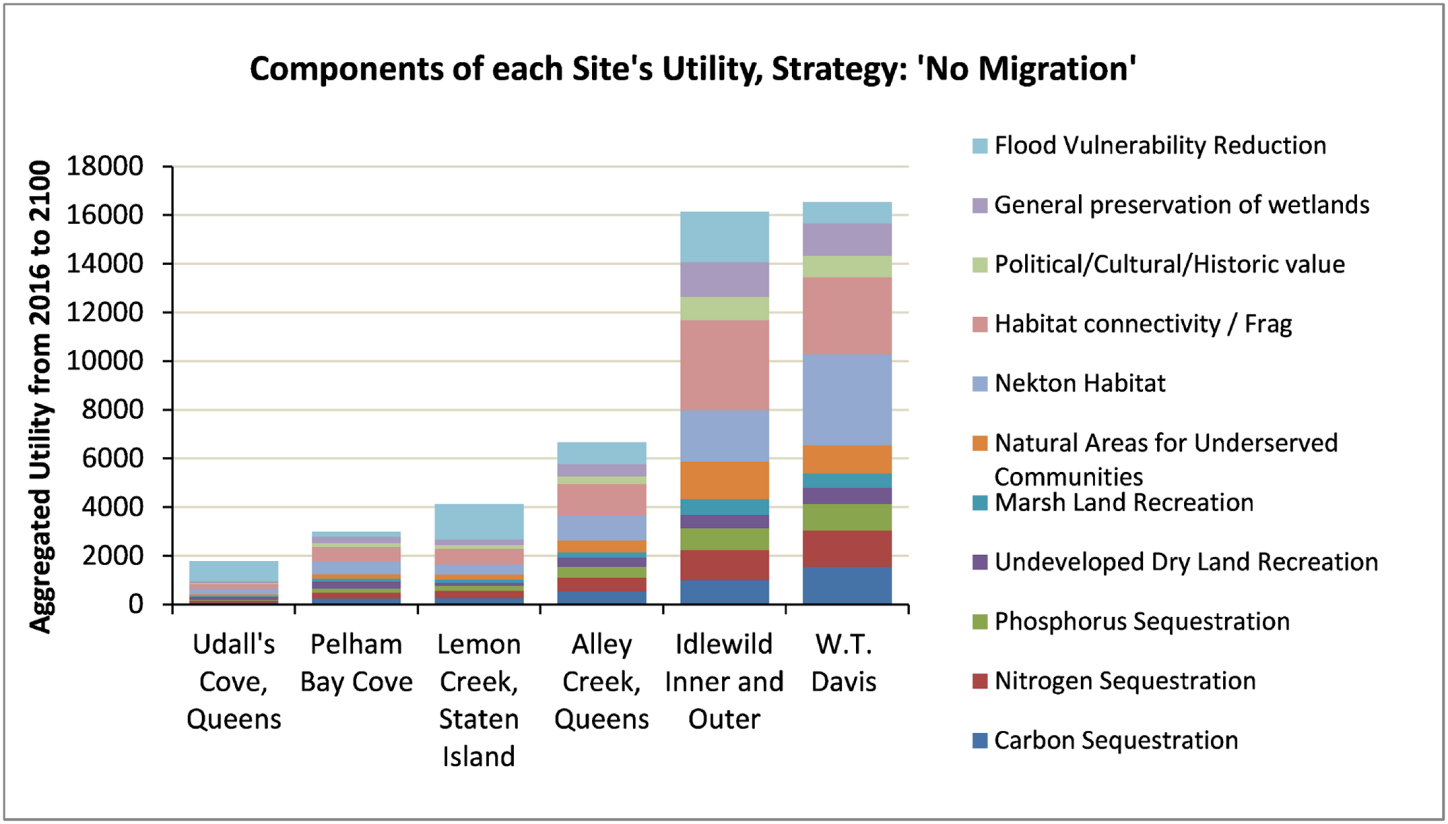
Constituents of each site’s aggregated utility from current marsh habitat (“no migration strategy”).

Fig 9 shows each of the components “*w*_*i*_ *S*_*i*_” of each site’s total utility “*W*” from Eq 3. The largest sites (Idlewild and W.T. Davis) are predicted to produce the largest benefits from habitat connectivity, nekton habitat, and nutrient sequestration. However, some smaller sites have significant benefits due to wave attenuation (Udall’s Cove and Lemon Creek).

## Discussion

This manuscript presents an integrated decision-support framework. This modeling product utilizes hundreds of marsh-fate simulations, which take into account both uncertainty in SLR and uncertainty in marsh response, and combines those results with an economic model describing stakeholder-derived ecosystem-service values. The framework produces cost/benefit metrics to inform optimal management decision making.

One result from the case studies produced in this project was that thin-layer deposition was not usually a cost effective strategy when aggregating benefits through the year 2100. The primary reason for this is that the SLAMM model, like most other marsh-fate models [31,32] includes a feedback between marsh elevation, inundation frequency, and the marsh elevation-change rate. When marsh lands increase in elevation they trap sediment less effectively. Over a decadal time scale the marsh surface of an elevation-augmented marsh and a no-action marsh are predicted to equilibrate. Thin-layer deposition also suffers in terms of cost effectiveness because it is not predicted to have any effect under some SLR scenarios examined within the model’s uncertainty analysis. Under low-SLR scenarios marsh losses are minimal, so there are minimal benefits to thin-layer deposition compared to the no-action scenario. Under the highest SLR scenarios, marshes are rapidly lost despite the initial 20 cm of sediment added to their surface, so again, differences from the no-action scenario are minimal. Despite these findings, thin-layer deposition may have an important role in keeping marshes viable under shorter planning horizons, or elevation capital may be further supplemented in future applications that are not included in these model runs. Additionally, the model did not try to optimize dredge placement for cost effectiveness. Instead, the model assumed that thin-layer deposition would occur on all regularly-flooded marshes that are accessible by barge or land.

**Fig 10:**
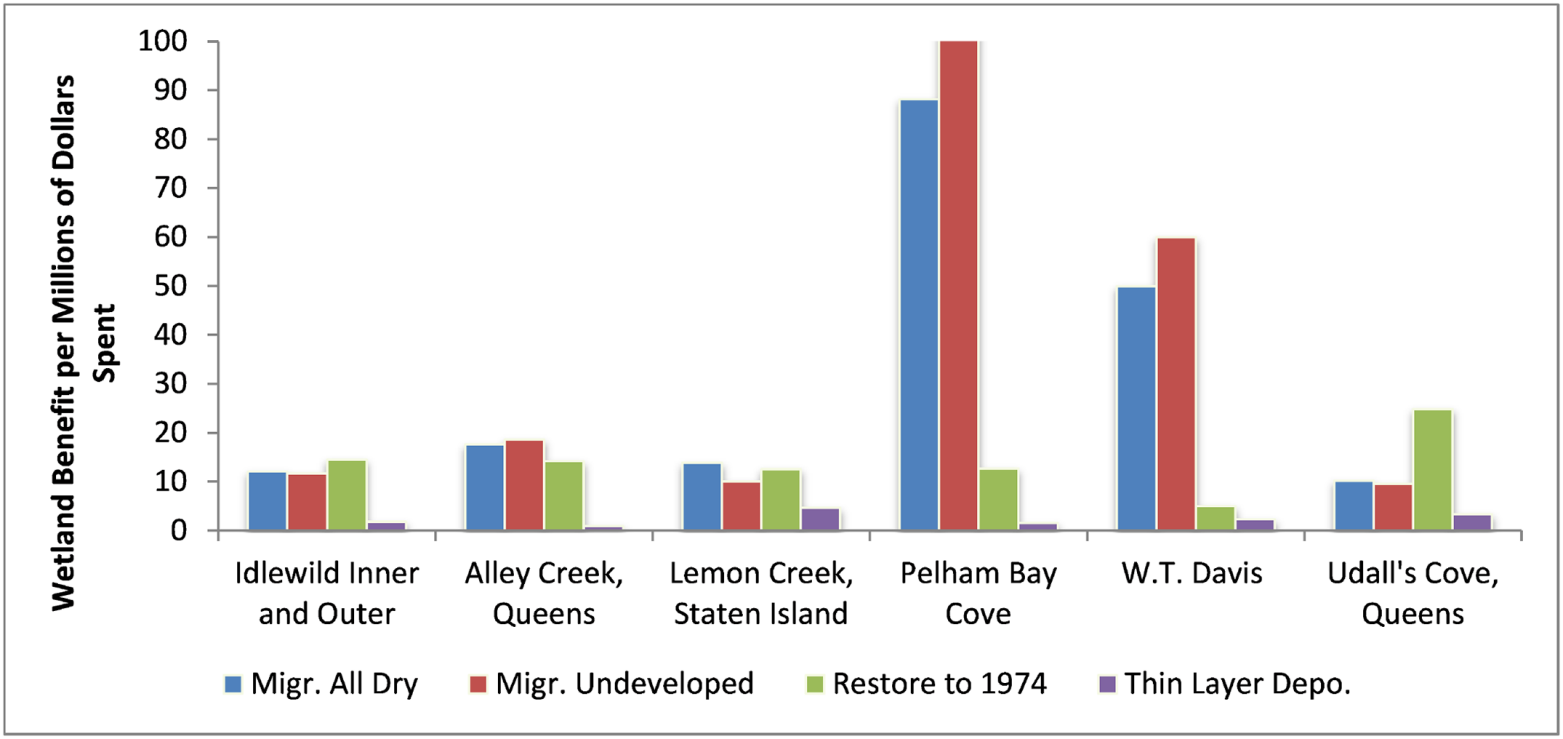
Aggregated wetland benefits predicted per million dollars spent (time interval 2085–2100).

Several DMMT user inputs can also play a significant role in the identification of optimal strategies. For example, choosing an alternative planning (time) horizon may change estimated optimal actions. Fig 5 suggested that restoration to 1974 marsh boundaries would be the most effective strategy for several marsh parcels when one considers cumulative benefits from current conditions to 2100 (e.g. Idlewild, Alley Creek, and Lemon Creek). However, if one exclusively looks at benefits provided from 2085–2100 (Fig 10), land acquisition becomes more cost effective in several of these sites. This result is because at that late date restored marsh parcels are predicted to be converted to open water at a higher rate and thus provide fewer benefits. The cost estimate for remedial alternatives at each parcel is also a sensitive model parameter.

It is also worth noting that the uncertainty-analysis results presented above combine projections under a range of plausible SLR scenarios, from more conservative (from 0.47 meters by 2100) to more extreme (2.01 meters by 2100). This distribution of future SLR greatly affects SLAMM projections in terms of probabilities of migration and marsh survival and therefore could significantly affect DMMT results. To examine this impact, model predictions were examined under SLR scenarios between 1.0 and 1.5 m by 2100. Limiting the results of the uncertainty analysis in this manner had minimal effects on the expected benefit per cost of marsh-adaptation strategies. This suggests that in this case study, with this set of priorities and cost assumptions, the selection of optimal management decisions seem to be robust despite SLR uncertainties. For the parcels considered here, prioritizing marsh migration at Pelham Bay Cove and W.T. Davis were predicted to be optimal adaptation strategies regardless both of SLR scenarios considered and time-horizons considered.

The case study presented here does not reflect a completed set of decisions using the DMMT tool, but instead provides insights on which marsh management actions might be most useful and cost effective given the cost and SLR assumptions within the model. As with any modeling approach there are limitations and simplifications; for example, land costs are not estimated on a tax-parcel basis but are estimated using an average cost per unit area based on current New York City land costs [29]. Furthermore, long-term results do not consider the potential for future management actions (such as additional thin-layer deposition) nor do they consider the timing of land acquisitions.

Ultimately the DMMT framework uses the latest spatial data to produce land-cover change projections while accounting for model and SLR uncertainties. Using these inputs, the framework considers stakeholder values and links them to ecosystem benefit outcomes in a manner that is a transparent, organized, and flexible. The result is a robust decision-support tool that allows stakeholders to plan adaptation strategies for marsh conservation and coastal community resiliency.
